# Pre-existing visual preference for white dot patterns in estrildid finches: a comparative study of a multi-species experiment

**DOI:** 10.1098/rsos.231057

**Published:** 2023-10-18

**Authors:** Ayumi Mizuno, Masayo Soma

**Affiliations:** Department of Biology, Faculty of Science, Hokkaido University, Sapporo, Hokkaido, Japan

**Keywords:** phylogenetic comparative methods, sensory bias, signal evolution, visual attention

## Abstract

The diverse characteristics of animal signal designs can be explained by the sensory bias hypothesis, which suggests that natural selection shapes sensory bias and preferences associated with signal design. Traditionally, this hypothesis has focused on female sensory biases and male sexual traits. However, considering shared sensory systems between males and females in non-sexual contexts, existing sensory bias possibly contributes to the evolution of shared social and sexual traits. Our previous studies on the family Estrildidae supported this idea. An evolutionary relationship probably existed between diet and white dot plumage, and a species of estrildid finches showed a visual preference for white dot patterns. To investigate this further, we examined hunger-related visual preferences using phylogenetic comparative methods and behavioural experiments. Specifically, we compared the gazing responses of 12 species of estrildids to monochromatic printed white dot and stripe patterns, considering their phylogenetic relationships. The results support our idea that the common estrildid ancestor had a hunger-related visual preference for white dot patterns. Subject species generally preferred white dots to stripes. Furthermore, males and females showed a similar preference towards dots. Our findings provide insights into the role of sensory bias in the evolution of mutual ornamentation.

## 1. Background

The evolutionary origin of signal design can be explained by the sensory bias hypothesis [1–3]. According to this hypothesis, natural selection acts on the presence of pre-existing sensory bias for a particular stimulus in females, facilitating the evolution of an ornamental trait in males. Particularly, there exist abundant examples of diet-driven female visual preferences contributing to the evolution of sexual ornaments in fish (e.g. [4–6]). For example, some Goodeinae species males have terminal yellow bands (TYB) on their tails to attract females with a visual sensitivity for foraging yellow worms [5]. Similarly, male guppies *Poecilia reticulata* also show a red dot pattern on their body surfaces to attract the attention of females with a foraging preference for red-colour fruits [4].

The crucial premise of the sensory bias hypothesis is the presence of pre-existing sensory bias. One can test such bias only by investigating if a particular sensory preference is shared among species of a focal taxonomic group regardless of whether they possess the signalling trait [2]. If one could directly assess sensory bias in the common ancestor, that would be ideal; however, behavioural experiments are never feasible for fossil species. Therefore, we can only infer the cognitive states of ancestral species based on the outcomes from behavioural experiments using extant species. In túngara frogs *Engystomops pustulosus* and related species within the genus *Engystomops*, females commonly share a preference for complex calls, irrespective of whether conspecific males produce such calls, which is considered one of the notable examples of pre-existing bias playing a role in signal evolution [1,7]. Similar findings have been reported for fiddler crabs *Uca beebei* [8], guppies *Poecilia reticulata* [4] and water mites *Neumania papillator* [9,10]. These studies appear to indicate the presence of pre-existing bias (e.g. [11]). Yet, some of these previous studies lacked the consideration of phylogenetic relationships among focal species, without which the estimation of the ancestral state could be biased by sampled species [12,13].

Furthermore, the previous empirical studies tended to overlook the possibility that the sensory bias hypothesis does not necessarily require sexual contexts. Although the idea can help to understand female mate choice and male ornamentation, its prediction can also be applied to the sensory bias shared between the sexes and its role in the evolution of social signals, such as status signalling outside the reproductive context. Indeed, the presence of sensory bias shared between the sexes has been suggested in túngara frogs [14] and guppies [4]. The role of sensory bias remains poorly understood in the evolution of mutual ornamentation.

**Figure 1.**
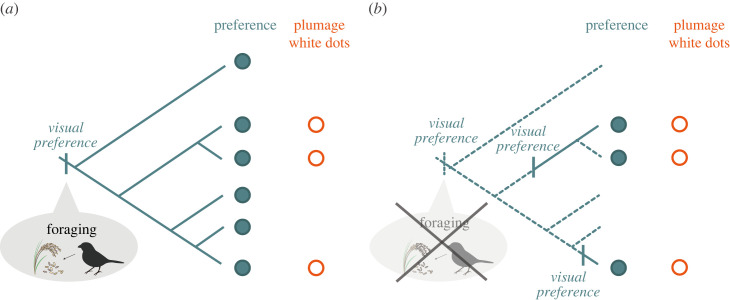
Predictions of the study regarding the sensory bias hypothesis and the evolution of plumage white dot patterns in estrildid finches. (*a*) If all extant estrildid finches show a visual preference for white dot patterns, the sensory bias hypothesis is supported as an explanation for the evolution of plumage dot patterns. (*b*) If the visual preference evolved in only specific extant species, the sensory bias hypothesis is inadequate in explaining the plumage dot evolution.

Our study aims to investigate whether shared sensory biases between males and females can explain the evolution of mutual ornamentation traits. In particular, we examine whether multiple species of estrildids (family Estrildidae) share and show similar visual preferences towards patterned stimuli, combining phylogenetic comparative methods and the experimental protocols previously used for star finches *Neochmia ruficauda* [15]. We hypothesize that visual preference is reflected in visual attention, which is measured based on the frequency of gazing [16–18]. Approximately 25% of 134 estrildid species are characterized by white dot plumage patterns, possibly with signalling functions [19–21]. Furthermore, both sexes often have similar white dot patterns on their plumage. Considering that estrildid species are mainly granivores [22,23], and that some species consume whitish gregarious round invertebrates (e.g. termites) [22], the visual preference for white dot patterns might also be important in foraging [24]. White dot plumage patterns were highly likely absent in the estrildid common ancestor [24]. However, no studies have tested whether sensory bias (visual preference) for dots existed in the ancestor of estrildids. If the pre-existing sensory bias existed in the ancestor, extant species of estrildids would possibly share a visual preference for white dots regardless of whether the species have white dot plumage patterns (figure 1*a*). In addition, if the visual preference was originally for foraging, a stronger visual preference for dots may be shown when they are hungry, and males and females may share similar preferences (figure 1*a*). Alternatively, when dot preference does not pre-exist, and evolves in particular extant species, among-species variations in diet (e.g. termite-eating; [24]) or plumage (i.e. presence of plumage dot patterns) would probably have associations with the preference (figure 1*b*).

## 2. Material and methods

### 2.1. Subjects

Adult individuals of 12 estrildid species (*N* = 95) were used. African silverbills *Lonchura cantans* (male: *n* = 3, female: *n* = 3), chestnut-breasted mannikins *L. castaneothorax* (male: *n* = 1, female: *n* = 3), chestnut munias *L. atricapilla* (male: *n* = 0, female: *n* = 2), double-barred finches *Taeniopygia bichenovii* (male: *n* = 3, female: *n* = 1), Gouldian finches *Chloebia gouldiae* (male: *n* = 3, female: *n* = 4), Java sparrows *Padda oryzivora* (male: *n* = 7, female: *n* = 10), painted firetail *Emblema pictum* (male: *n* = 0, female: *n* = 1), plum-headed finches *Neochmia modesta* (male: *n* = 1, female: *n* = 5), red-cheeked cordon-bleus *Uraeginthus bengalus* (male: *n* = 7, female: *n* = 5), star finches *N. ruficauda* (male: *n* = 15, female: *n* = 11), white-headed munias *L. maja* (male: *n* = 2, female: *n* = 0) and zebra finches *T. guttata* (male: *n* = 4, female: *n* = 4), were obtained from several local breeders or kept in Wada or Soma laboratory in Hokkaido University. We only included adult birds without breeding experience. Each bird was identified with a unique combination of coloured leg rings. Birds were kept in unisex cages (1.0 mm wide fences with 12.0 mm spacing), visible to each other, on a 12 : 12 h light : dark schedule (lights on 08.00–20.00) at approximately 25–26°C and 50–60% humidity. They were given a finch seed mixture, cuttlebone, water and fresh green vegetables daily unless tested under food-deprived conditions. Each bird was tested in the experiments conducted between May and June 2019, January and June 2020, or November 2021 and March 2022.

### 2.2. Experimental procedure

The behavioural experiments were conducted using the same methods described in Mizuno & Soma [15]. Briefly, each experimental bird was singly placed in an experimental cage and presented for an hour with pairs of stimuli, which were paper printed with white dots (diameter 2.0 mm) or stripes (width 2.0 mm). All experiments were conducted in an experimental room under constant and controlled light conditions. We lighted from above the experimental cage using an LED light stand (1100 lm m^−^^2^), and the room was illuminated by LED lights (1880 lm m^−2^). During the experimental period, their behaviours were recorded on a video camera (GC-PX1, Victor, Tokyo, Japan). The experiment was carried out over 2 days after 24 h habituation. On day 1, the experiment was conducted under the food-deprived condition to examine hunger-related preference. On day 2, the experiment was conducted under the food-supplied condition to test the hunger-neutral preference. The stimulus positions were reversed between days 1 and 2 to control for side preferences. From video recordings, we focused on gazing behaviour as an index of visual preference [25,26] towards each stimulus during each condition for every subject bird. The data of gazing frequency on days 1 and 2 were analysed separately under the assumption that they reflect hunger-related and hunger-neutral responses, respectively. All videos were scored blindly by the same observer (A.M.).

### 2.3. Statistical analysis

#### 2.3.1. Phylogenetic comparative analysis

To consider the limited sample size (number of individuals) and lack of balanced sex data for some species, for the below-mentioned analyses, we used both full-species data, including all subject species (*n* = 12 species; 95 individuals), and subset data excluding species without behavioural data on one sex (*n* = 9 species; 92 individuals) except when otherwise stated [27]. As a dependent variable, we used gazing frequency towards the two stimuli (dots versus stripes) to examine whether the proportion of subjects' responses to each stimulus deviated from those expected by chance.

To test taxon-wide dot preference in estrildid finches, the model was applied to full and subset data of gazing frequency towards dot versus stripe stimuli using a Bayesian linear mixed model by the R package ‘MCMCglmm’ [28] with binomial distribution with consideration of their phylogenetic relationships. In the model, we included the frequency of gazing at the two stimuli as a bound response variable and condition (food-deprived versus food-supplied) as an explanatory variable. The global intercept was removed from the model to allow trait-specific intercepts to be estimated. Phylogenetic information was entered as a random effect to control for possible phylogenetic non-independence. A phylogenetic tree, obtained from Olsson and Alström [29] was modified using the force.ultrametric function of the R package ‘phytools’ to include only focal species [30] and use all analyses. In the model, the prior was set as [R = list(V = 1, nu = 0.002), G = list(G1 = list(V = 1, nu = 1, alpha.mu = 0, alpha.V = 100))], and a chain was run for 5 000 000 iterations, sampling every 500 iterations after a 1000 burn-in. To facilitate the interpretation of the model results, we converted the estimated posterior means and 95% confidence intervals to percentages using the inverse logit function from package ‘arm’ [31]. The results before conversion are summarized in table 1, electronic supplementary material, tables S1 and S2.

**Table 1.** Summary of MCMCglmm under the two conditions, showing the difference in gazing responses between the two stimuli (white dot versus stripe). The italic typeface is used when a 95% confidence interval (CI) does not contain zero; thus, it can be interpreted as an existing significant difference in gazing frequency towards the dots.

dataset	condition	posterior mean	95% CI	*p*MCMC
(a) full-species	*food-deprived*	*0.467*	*(0.030, 0.860)*	*0.039*
	food-supplied	0.247	(−0.171, 0.658)	0.195
(b) subset	*food-deprived*	*0.514*	*(0.254, 0.777)*	*0.004*
	*food-supplied*	*0.360*	*(0.096, 0.630)*	*0.016*

The factors influencing the difference in visual preference between the white dot and stripe pattern stimuli were also examined. Specifically, the effects of sex, diet (termite-eating: whether subject species consume whitish round tiny invertebrates, such as termites or ant eggs), and the presence/absence of white dot plumage patterns on gazing frequency towards the two stimuli were tested by adding explanatory variables to the above-mentioned intercept-only MCMCglmm. The data on termite-eating and the presence/absence of white dot plumage patterns were based on Soma & Garamszegi [21] and Mizuno & Soma [24], respectively. As the evolutionary correlation between them was shown by Mizuno and Soma [24], two sets of MCMCglmm, one with sex and termite-eating as explanatory variables and the other with sex and the presence of white dots, were prepared, instead of full models including all three explanatory variables. After running MCMCglmm, we applied visually inspecting plots of MCMC chains and the Gelman–Rubin statistic [32], which confirmed model convergence and good mixing of the chains.

Finally, we performed ancestral state reconstruction using tips and internal nodes model estimates from the above first MCMCglmm model with the ‘ape’ and ‘phytools’ packages [30,33]. We did not adjust the alpha levels of these tests because adjusting them in cases with limited sample sizes increases the possibility of type II errors [34,35]. All analyses were conducted in R 4.2.3 [36]. Raw data, analysis script (R code) and supplementary material videos are available at [27].

## 3. Results

### 3.1. Visual preference

**Figure 2.**
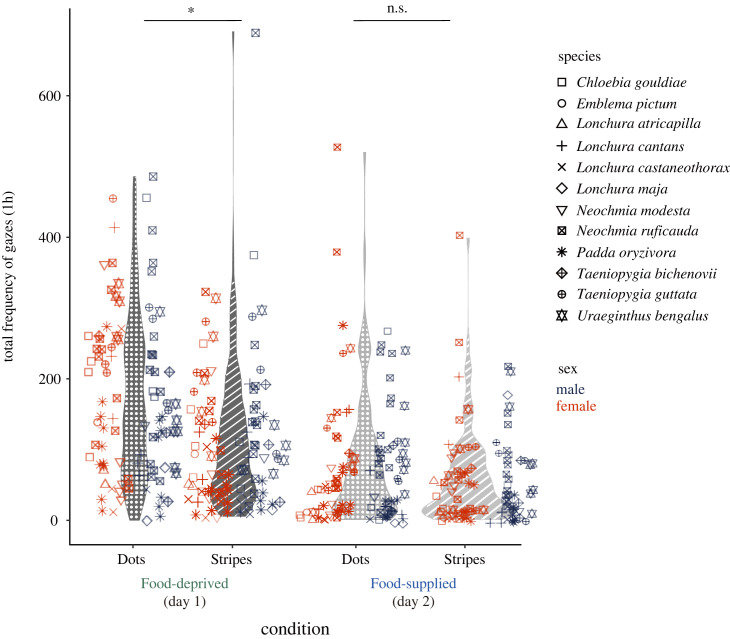
The total frequency of gazes toward white dot and stripe stimuli under the food-deprived and food-supplied conditions based on the full-species dataset. Each plot represents a different subject, and different plot shapes correspond to different subject species.

In the full-species data, subject birds showed a significant preference for white dot patterns under the food-deprived condition, with an estimated proportion of approximately 61.5% (95% CI = 50.7% to 70.3%, *p*MCMC = 0.039; figure 2, table 1 (a)), which is higher than the chance level (50%), but not under the food-supplied condition (posterior mean = 56.1%; 95% CI = 45.7% to 65.9%, *p*MCMC = 0.195; figure 2, table 1 (a)). In the subset data, subject birds showed a statistically significant visual preference for dots over stripes under the food-deprived condition, with an estimated proportion of approximately 62.7%, which is higher than the chance (95% CI = 56.4% to 68.6%, *p*MCMC = 0.002; table 1 (b)), and this did not change under the food-supplied condition (posterior mean = 59.0%; 95% CI = 52.6% to 65.4%; *p*MCMC = 0.015; table 1 (b)).

The models including explanatory variables sex and termite-eating, as well as those including sex and dot pattern plumage, indicated no significant sex differences in visual preference towards dots over stripes under the food-deprived condition (full-species data; sex and termite-eating model's posterior mean = 41.6%, 95% CI = 33.2% to 50.1%, *p*MCMC = 0.061; sex and white dot plumage patterns model's posterior mean = 41.5%, 95% CI = 38.9% to 64.9%, *p*MCMC = 0.053; figure 2, electronic supplementary material, table S1, subset data; electronic supplementary material, table S2). The results obtained from the food-deprived condition showed the same trend (electronic supplementary material, tables S1 and S2). Visual preference was not significantly affected by termite-eating or the presence of white dot plumage patterns either under the food-deprived or food-supplied conditions in the full-species and subset data (electronic supplementary material, tables S1 and S2).

### 3.2. Ancestral state reconstructions of visual preference

**Figure 3.**
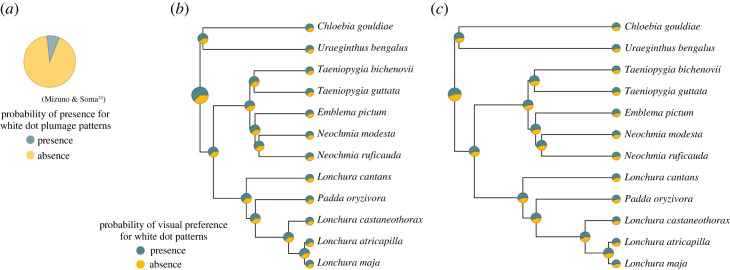
(*a*) The pie chart shows the likelihood ancestral state of the presence or absence of white dot plumage patterns inferred based on all 134 estrildid extant species (ancestral state reconstruction using the maximum likelihood method, adapted from [24]). Ancestral state reconstructions based on MCMCglmm of (*b*) hunger-related and (*c*) hunger-neutral visual preferences for white dot patterns using full-species data of this study (*n* = 12 species, 95 individuals).

The ancestral states of hunger-related white dot visual preference were likely to present in estrildid common ancestor because the probability of root node is 62.2% (figure 3*b*). Hunger-neutral visual preference for white dot patterns was estimated to be unclear in the common estrildid ancestor, according to the estimated ancestral state (root node: the presence of visual preference for white dots 52.0%; figure 3*c*).

## 4. Discussion

Our findings support our idea that the pre-existing sensory bias for white dot patterns existed in the estrildid common ancestor (figure 1*a*) and not only evolved in specific extant species (figure 1*b*). We revealed that both sexes of estrildid finches generally preferred to look at white dots over stripes (figure 2, table 1), regardless of whether the species had white dot plumage patterns or termite diet (electronic supplementary material, tables S1 and S2). Additionally, it can be inferred that the common ancestor of estrildid finches could have paid more attention to white dots than white stripes when they were hungry (figure 3*b*). The obtained results were based on a relatively small number of species and may have less robustness. However, they can be considered as reflecting taxon-wide visual preference because the subject species covered major genera of the family Estrildidae. Some species were from the same genus, but our analyses considered them using phylogenetic comparative methods. Using simple abstract pattern stimuli, we minimized the possibility that subjects' responses were influenced by their prior experience and assumed that they reflected intrinsic visual preference. Our idea that white dot preference in estrildids is a shared ancestral trait might be more strongly supported if we could have adjusted for prior visual experience of the subject birds in the study (e.g. for the presence of cage fences, feeding of round seeds or thin worms, or interaction with conspecifics or related species). The findings derived from the subset data, which indicate that estrildid finches tend to gaze at white dot patterns even in hunger-neutral conditions (table 1 (b)), provide additional support for the idea that these finches possess an intrinsic visual preference for such patterns. The visual preference observed in extant species suggests that a pre-existing sensory bias exists in the ancestor of estrildids.

The preference for the white dot pattern stimuli was significant only when the birds were hungry (figure 2, table 1), meaning that the visual preference is associated with foraging, consistent with previous studies that indicated the role of foraging-related visual preference in the evolution of male ornamental traits (e.g. [4,5,9,10]). The ancestral state reconstruction also showed a moderate probability of having hunger-related visual preference (figure 3*b*). Given birds’ excellent visual acuity and colour vision [37], we presumed that estrildid finches would not mistake the abstract white dot pattern stimulus for their food. Estrildids' visual preference for white dot patterns could probably be associated with searching for grain seeds on the ground, as all estrildid species are granivores [22,23].

Consistent with the prediction that both sexes may share similar preferences, there was no statistically significant difference in the visual preferences of males and females between white dots and stripes (electronic supplementary material, tables S1 and S2). Considering this finding and that the common ancestor of estrildid finches was unlikely to have white dot plumage patterns (figure 3*a*; [24]), it is in line with our idea that the visual preference for white dots is associated with the evolution of male and female ornamental plumage dots. The present finding showed that pre-existing sensory bias would be shared in species where the signal trait is shared between males and females and plays a role in the evolution of sexual and social signalling traits. Particularly, it would be necessary for estrildid finches to have signal traits that are likely to attract the attention of other conspecifics of both sexes. They show social and sexual displays at a close distance, often perching side by side [20,22,38,39]. Moreover, conspicuous dots on their flanks could serve as clues for species recognition. Estrildid finches occasionally create mixed-species flocks and forage for food together [22,23]. The presence of white dot plumage patterns may help to find their own species from other species. Alternatively, white dot patterns possibly facilitate the detection of conspecifics at far distances. The visual preference of male and female estrildid finches could be crucial in both sexual and social contexts, considering that over 25% of these finches exhibit white dot plumage patterns of similar conspicuousness, typically located on their flanks [21,24]. In our earlier study, estrildid species with conspicuous white dots on their plumage had a strong visual preference for dots regardless of food availability [15]. This finding also supports that such species would show visual preference under the foraging and communication context.

Our pioneering study represents the first exploration of the sensory bias hypothesis in avian signalling traits shared between sexes, offering new insights into the evolution of social and sexual signals in birds. It is also worth noting that our study is among the few that combine phylogenetic comparative methods with behavioural experiments. Previous studies have investigated various signal characteristics used by birds, such as sounds, dances and smells (e.g. [38,40–46]), as well as bird plumage colours and patterns (e.g. [47–49]). Yet the factors associated with the evolutionary origins of these traits remain unknown. The sensory bias hypothesis can provide one possible explanation for the origin of signalling trait evolution in birds. Our findings can extend the applicability of this hypothesis to a broader range of taxa and provide valuable insights into the evolutionary origins of both social and sexual signals. Lastly, the present study enhances our understanding of visual preferences in estrildids and sheds light on how birds perceive the world. While we used only two stimuli, further investigation using various stimuli could provide a more comprehensive understanding of visual preferences in estrildids and other avian species.

## Data Availability

Data and R code are available via Open Science Framework: https://doi.org/10.17605/OSF.IO/CPEBF [50]. The supplementary results are provided in the electronic supplementary material [51].
